# Investigation of the Effects of Various Severe Plastic Deformation Techniques on the Microstructure of Laser Powder Bed Fusion AlSi10Mg Alloy

**DOI:** 10.3390/ma16237418

**Published:** 2023-11-29

**Authors:** Przemysław Snopiński, Krzysztof Matus, Ondřej Hilšer

**Affiliations:** 1 Department of Engineering Materials and Biomaterials, Silesian University of Technology, 18A Konarskiego Street, 44-100 Gliwice, Poland; 2 Materials Research Laboratory, Silesian University of Technology, 18A Konarskiego Street, 44-100 Gliwice, Poland; krzysztof.matus@polsl.pl; 3 Faculty of Mechanical Engineering, VSB-TU Ostrava, 17. listopadu 2172/15, Poruba, 70800 Ostrava, Czech Republic; ondrej.hilser@vsb.cz

**Keywords:** AlSi10Mg, ECAP, KoBo extrusion, multi-axial forging, microstructure

## Abstract

In this paper, we present a complete characterization of the microstructural changes that occur in an LPBF AlSi10Mg alloy subjected to various post-processing methods, including equal-channel angular pressing (ECAP), KoBo extrusion, and multi-axial forging. Kikuchi transmission diffraction and transmission electron microscopy were used to examine the microstructures. Our findings revealed that multi-axis forging produced an extremely fine subgrain structure. KoBo extrusion resulted in a practically dislocation-free microstructure. ECAP processing at temperatures between 100 °C and 200 °C generated moderate grain refinement, with subgrain diameters averaging from 300 nm to 700 nm. The obtained data highlighted the potential of severe plastic deformation as a versatile method for tailoring the microstructure of the AlSi10Mg alloy. The ability to precisely control grain size and dislocation density using specific SPD methods allows for the development of novel materials with ultrafine-grained microstructures that offer the potential for enhanced mechanical and functional properties.

## 1. Introduction

Aluminum has many advantages, including low density, high specific strength, excellent oxidation resistance, high thermal conductivity, and high electrical conductivity [1]. It is an ideal structural material for lightweight automotive, transportation, and aerospace components. However, the most interesting transformative change in the utilization of aluminum is its integration into the laser powder bed fusion (LPBF) process. In recent years, among the various known combinations of aluminum alloys available, silicone-containing alloys such as AlSi10Mg and Al12Si have been the most explored. Specifically, AlSi10Mg has attracted the most attention due to its desirable properties, such as high corrosion resistance, superior strength, and excellent dynamic toughness [2]. In addition, this alloy possesses excellent processability, flowability, and a narrow solidification range [3]. The near-eutectic composition of AlSi10Mg allows the production of volumetrically dense parts free of solidification cracks [4]. Furthermore, the high Si content contributes to the improvement of mechanical properties, making AlSi10Mg LPBF components suitable for various lightweight applications.

Numerous articles [5,6,7,8] have described the excellent mechanical properties of LPBF Al-Si alloys, which stem from their unique microstructure. It is well known that LPBF Al-Si alloys have an extremely heterogeneous microstructure. These microstructural heterogeneities can be divided into four different levels: (i) melt pools, (ii) heterogeneous grains, (iii) cell structures, and, in some cases, (iv) nanoprecipitates. In particular, the nanoscale cell structure decorated with Si particles is crucial for strengthening LPBF AlSi10Mg alloys by effectively blocking the dislocation motion at the cell boundaries [9]. Furthermore, the unique cell structure in the LPBF alloys has a significant influence on the deformation behavior. For example, if the cellular morphology is partially or fully eliminated by annealing or other heat treatments, the work hardening rate may decrease due to the loss of dislocation–obstacle interactions [10,11,12]. Therefore, stress-relieved [13], T6-treated [14], or hot isostatic pressed (HIP) [15] components generally exhibit lower yield strength than the as-built parts [16], which is mainly due to the coarsening of the microstructure and spheroidization of the Si eutectic network [17].

To improve the properties of Al-Si alloys, researchers have investigated the effectiveness of surface modification post-processing. For example, Maamoun et al. [18] used the shot peening technique to improve the surface integrity and mechanical properties of the LPBF parts. This approach enhanced surface hardness, eliminated surface defects, and refined the microstructure. In addition, the surface properties were further improved via the application of friction stir processing (FSP). This resulted in a more uniform microstructure with a better distribution of Si particles in the aluminum matrix. However, FSP also reduced the microhardness of the as-built sample from 120 HV to 76 HV [19]. Some studies have investigated the effects of ultrasonic peening treatment (UPT) on LPBF AlSi10Mg alloys [20]. The reported data showed that UPT significantly increased Vickers hardness (it reached approx. 140 HV, compared to the initial value of 115 HV). Similarly, Maleki et al. [21] compared laser shock peening and ultrasonic nanocrystalline surface modification for the same alloy. Improved hardness, yield strength, and tensile strength, with some retention of ductility, were reported. Importantly, the application of the above-mentioned techniques primarily refined the microstructure in the near-surface region, leaving the bulk material’s original grain size distribution mostly unaffected.

Recent studies have examined the effects of severe plastic deformation (SPD) on the microstructure and properties of AlSi10Mg produced by LPBF [22,23,24]. SPD refers to a series of metal-forming techniques in which very high strains are applied to the material without significantly changing its shape. SPD techniques can be used to produce metallic materials with ultrafine grains (UFG), typically <500 nm in size, or even grains in the nanometer range, which significantly improves yield strength, and wear and corrosion resistance [25]. These processes include high-pressure torsion (HPT) [26], accumulative roll bonding (ARB) [27], twist extrusion (TE) [28], equal-channel angular pressing (ECAP) [29], and others [30,31,32]. For LPBF aluminum alloys, considerable efforts have been made to investigate the effects of ECAP and HPT processing on microstructure and mechanical properties [22,33,34]. The reports showed that post-processing of LPBF parts by SPD provided both high strength and improved ductility. For example, a representative study [22] showed that four ECAP passes resulted in significant grain refinement of the microstructure of the LPBF AlSi12 alloy, leading to a significant increase in the yield strength from 270 MPa to 420 MPa while maintaining a reasonable ductility of 5.9%.

In this study, we analyzed the microstructural evolution of an LPBF AlSi10Mg alloy subjected to various deformation techniques. Our research compared the microstructures obtained with the studied deformation techniques and determined which technique gave the largest grain refinement. In particular, we applied transmission electron microscopy and Kikuchi transmission diffraction (TKD) to shed light on microstructural evolution.

One of the key new aspects of our research was TKD characterization, which provided improved spatial resolution and allowed for the examination of LPBF materials at a finer scale. Additionally, it played a central role in the unraveling of the complex dislocation networks at the cell boundaries. These networks are known to be instrumental in improving material properties by impeding the movement of newly formed dislocations during deformation [35].

Unfortunately, despite the critical importance of understanding deformation mechanisms and deformation-related defects such as dislocations and grain boundaries, little work has been published. Our study bridges this knowledge gap by contributing to the improvement of the mechanical strength of LPBF aluminum alloys and unlocking the full potential of these advanced materials. Our work is a novel and versatile approach to the study of LPBF materials, incorporating advanced characterization techniques and post-processing methods that collectively represent a significant advance in the field.

This research examines the influence of deformation temperature and various processing methods, such as ECAP, KoBo extrusion, and multi-axial forging, on microstructural evolution. Our findings revealed that SPD shows great promise in the fabrication of novel materials with ultrafine-grained microstructures, which could potentially exhibit exceptional mechanical and functional properties.

## 2. Materials and Methodology

In this study, gas-atomized spherical AlSi10Mg alloy powder was employed to produce bulk samples with an LPBF machine. A statistical experimental design was applied to determine the optimal process parameters (scanning speed, laser power, layer thickness, and hatch spacing) that reduced defects (pores or cracks). Then, the mechanical properties of the samples were tested to select the samples with the highest yields and tensile strengths. The following process parameters were chosen for the production of the working specimens with a porosity level of 0.25% [36]:

- Laser power: 175 W;
- Scanning speed: 1.4 m/s;
- Hatch distance: 0.02 mm;
- Scanning strategy: bidirectional.

**Figure 1:**
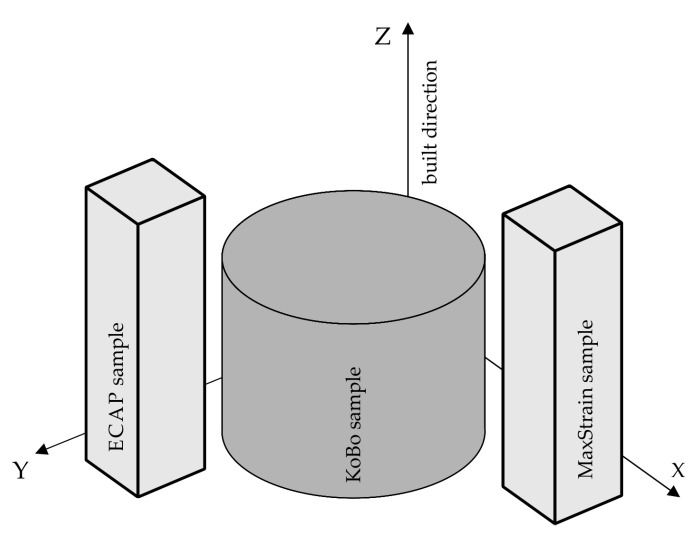
The manufacturing strategy adopted for the printing of AlSi10Mg parts.

The samples were printed along the z-direction, parallel to the direction of the laser beam. Figure 1 shows a schematic illustration of the manufacturing strategy adopted for printing AlSi10Mg samples.

Following the SLM process, the samples were pre-machined to the desired shapes and then subjected to a series of post-processing procedures to investigate the effects of different starting microstructures and plastic deformation methods on the microstructural evolution. In this study, five different post-processing conditions are examined for comparative purposes:

- Annealing at 320 °C for 9 min and one ECAP pass at 100 °C—labeled as sample HT320E100;
- Annealing at 320 °C for 9 min and one ECAP pass at 130 °C—labeled as sample HT320E130;
- Annealing at 280 °C for 9 min and one ECAP pass at 200 °C—labeled as sample HT280E200;
- As-built samples subjected to multi-axial forging at 100 °C—labeled as sample MAF100;
- As-built samples subjected to KoBo extrusion.

**Figure 2:**
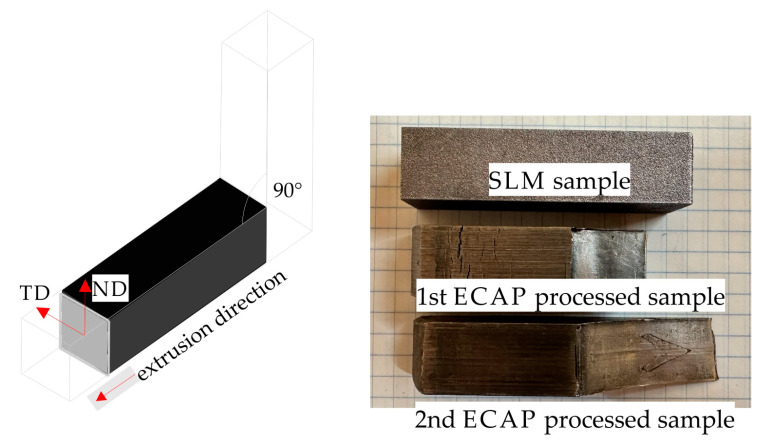
Schematic illustration of the ECAP process.

The ECAP experiment was performed using the LabTest 5.2000CT hydraulic press. The internal dimensions of the ECAP die were 14.25 × 14.25 × 60 mm. Therefore, the specimens for the ECAP experiment were cut to 14.20 × 14.20 × 50 mm and pressed once through a 90° die, which introduced an equivalent strain of ε ≈ 1, as shown in Figure 2. In order to reduce friction and the ECAP channel, the sample surface was covered with a thin layer of high-temperature grease.

**Figure 3:**
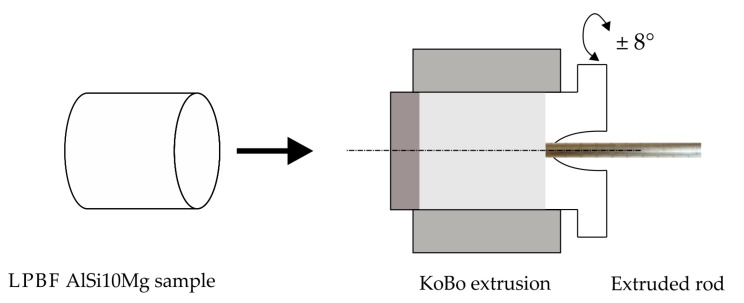
The schematic illustration of KoBo process.

The KoBo extrusion process (Figure 3) was carried out on a custom-made hydraulic press using the following experimental parameters:

- Punch speed of 0.2 mm/s;
- Die rotation angle of ±8°;
- Frequency of 5 Hz;
- Extrusion ratio λ = 225;
- Maximal measured temperature close to the extrusion die (~280 °C);
- Sample cooling—room-temperature water.

**Figure 4:**
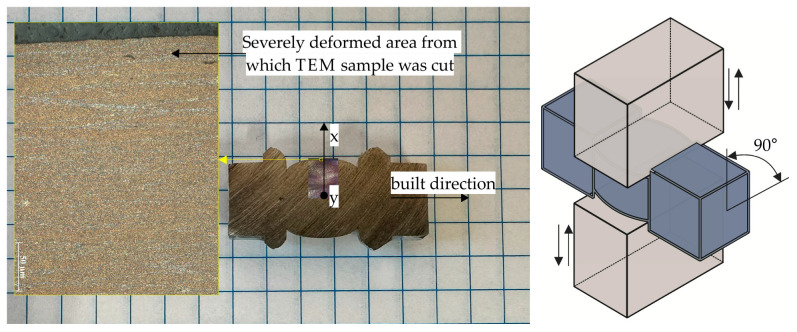
The AlSi10Mg alloy sample after MAF process and a schematic illustration of multi-axial forging process.

The multi-axial forging (MAF) process was carried out using MAXStrain Gleeble (Dynamic Systems Inc., Poestenkill, NY, USA) equipment. A 27 mm long specimen with a 10 × 10 mm rectangular cross-section and deformable region of 10 × 10 × 11 mm was pre-machined from LPBF material for MaxStrain experiments. The sample was then subjected to four compression cycles in an open die along the x and y axes at 100 °C and a strain rate of 10 s^−1^. The first and second compression cycles deformed the sample to ε = ~0.1 (1 mm compression). The third and fourth compression cycles deformed the sample up to ε = ~0.2 (2 mm compression). This meant that the total imposed strain was equal to ε = ~0.3, according to the following equations:

(1) $$\epsilon_{x}=\frac{∆L_{x}}{{L0}_{x}}$$

(2) $$\epsilon_{y}=\frac{∆L_{y}}{{L0}_{y}}$$

where ε_x_ is the strain in the x-direction, ΔL_x_ is the change in length in the x-direction, L0_x_ is the initial length in the x-direction, ε_y_ is the strain in the y-direction, ΔL_y_ is the change in length in the y-direction, and L0_y_ is the initial length in the y-direction. Figure 4 shows the sample cross-section after the MAF process.

A focused ion beam (FIB) was used to prepare thin lamellas for electron microscopy observations. The lamellae fabrication process involved iterative Ga ion milling steps, which resulted in a final sample with a thickness of approx. 120 nm. In the case of the ECAP and KoBo samples, cuts were made along the extrusion direction, while for the MAF100 sample, they were made along the compression direction. A single TEM lamella was prepared under each analyzed condition.

The microstructures were then characterized in detail using a transmission electron microscope (TEM) and a scanning electron microscope (SEM).

For SEM characterizations, the samples were prepared according to standard metallographic techniques (grinding with SiC papers and polishing with 6, 3, and 1 µm diamond pastes). To reveal the microstructures, the samples were etched for 30 s using Keller’s reagent.

For TEM characterizations, an S/TEM Titan 80-300 FEI (Hillsboro, OR, USA) microscope was used. Electron diffraction images were further analyzed using Digital Micrograph and CrysTBox (Crystallographic Toolbox) software (version number 1.10).

EBSD TKD observations were performed on a Zeiss Supra 35 (Carl Zeiss NTS GmbH, Oberkochen, Germany) field emission SEM. The TEM lamellas were held at a net angle of 10° with respect to the horizontal by a custom-made holder. The operating distance was determined to be 7.5 mm. The electron beam energy was held constant at 20 kV during analysis. The TKD maps were acquired using a 20 nm step size. Then, the TKD orientation maps were post-processed with ATEX software (version number 4.09), which provided detailed information on grain boundary characteristics.

## 3. Results

### 3.1. Microstructure Prior to Deformation

#### 3.1.1. SEM Microstructures

**Figure 5:**
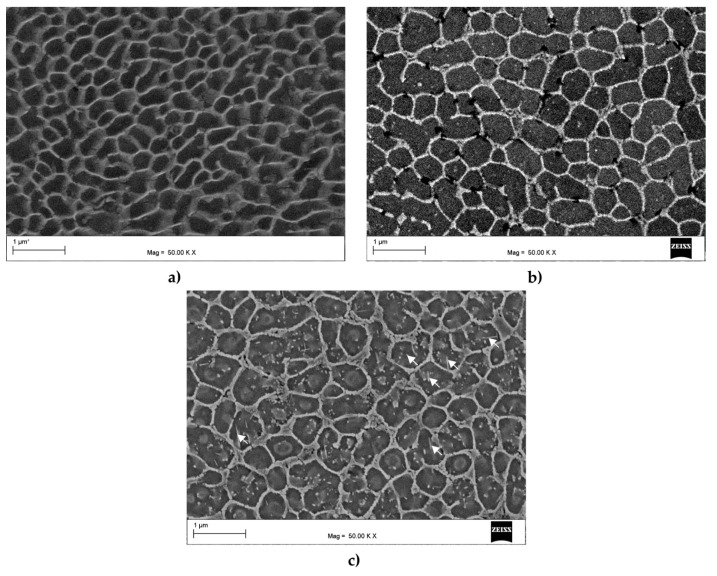
SEM microstructures of the AlSi10Mg alloy prior to deformation: (**a**) as-built; (**b**) annealed at 280 °C for 9 min (HT280); (**c**) annealed at 320 °C for 9 min (HT320).

Figure 5a–c show the microstructure within the core of the melt pool for the as-built, HT280, and HT320 samples. In the as-built sample (Figure 5a), the microstructure typically consisted of α-Al cells, surrounded by a continuous eutectic Si network with a measured average cell size of about approx. ~0.36 µm. Since the LPBF microstructure was highly metastable [37], it continued to evolve during the heat treatment process. As shown in Figure 5b,c, the cells in the annealed samples were coarser than the as-built sample. In the HT280 and HT320 samples, the measured average cell sizes were approx. ~0.55 µm and 0.58 µm, respectively. The heat treatment also led to a partial change in the continuity of the Si network, making it more fragmented compared to the as-built sample. Furthermore, the increased diffusivity of Si atoms resulted in the precipitation of Si particles from the supersaturated solid solution [38]. These precipitates were located within the α-Al cells (as indicated by the white arrows in Figure 5c). In general, the observed evolution of the microstructure after heat treatments was consistent with numerous experimental results reported in recent years [39,40,41].

#### 3.1.2. Grain Features and Crystallographic Orientation

**Figure 6:**
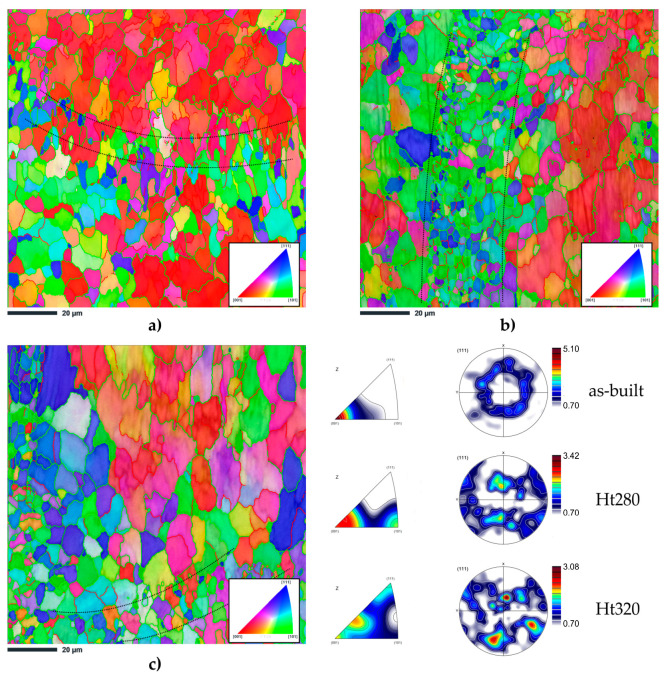
SEM microstructures of the AlSi10Mg alloy prior to deformation: (**a**) as-built; (**b**) annealed at 280 °C for 9 min (HT280); (**c**) annealed at 320 °C for 9 min (HT320). In the IPF maps, low-angle boundaries 2° < θ < 15° are represented via red lines, whereas high-angle boundaries 15° < θ < 65° are represented via green lines.

To obtain a better understanding of the grain structure, EBSD analysis was performed. Figure 6 shows the inverse pole figure (IPF) and pole figure (PF) maps of the as-built, HT280, and HT320 samples.

Figure 6a shows an obvious grain distribution with the preferred <001> orientation, while the (111) pole figure of the as-built sample had a fiber texture typically observed in the LPBF AlSi10Mg alloy.

After annealing at 280 °C for 9 min, the preferred grain orientations and the microtextures displayed a slight change. The IPF color map showed that in this sample, grains with orientations of <001> and <101> coexist (Figure 6b). The (111) pole figure indicated the formation of a texture dominated by the Cube and Goss components.

The sample annealed at 320 °C for 9 min showed a markedly different microtexture (Figure 6c). The (111) pole figure confirmed further transformation to a Cube and Goss texture, which is a typical recrystallization texture in aluminum alloys [42].

The EBSD orientation maps also revealed a subtle increase in grain coarsening as the annealing temperature increased. According to the statistical data given in Table 1, the average grain sizes of 3.9 µm, 4.2 µm, and 4.6 µm corresponded to the as-built, HT280, and HT320 samples, respectively.

The grain boundary misorientation, another crucial crystallographic characteristic, was analyzed. It was found that high-angle grain boundaries (HAGBs) dominated each of the studied samples. Specifically, the HAGB and low-angle grain boundary (LAGB) number fractions of the as-built sample were 79.8% and 20.2%, respectively. After annealing at 280 °C for 9 min, the HAGB number fraction increased to 86.4%, and the LAGB number fraction decreased to 13.6%. A similar trend was observed for the sample annealed at 320 °C for 9 min. The HAGB number fraction increased to 86.3%, and the LAGB number fraction decreased to 13.7%. The low fraction of the LAGBs could be associated with the occurrence of the recrystallization phenomena [43].

### 3.2. ECAP-Processed Samples

#### 3.2.1. Kikuchi Transmission Diffraction Analysis

**Figure 7:**
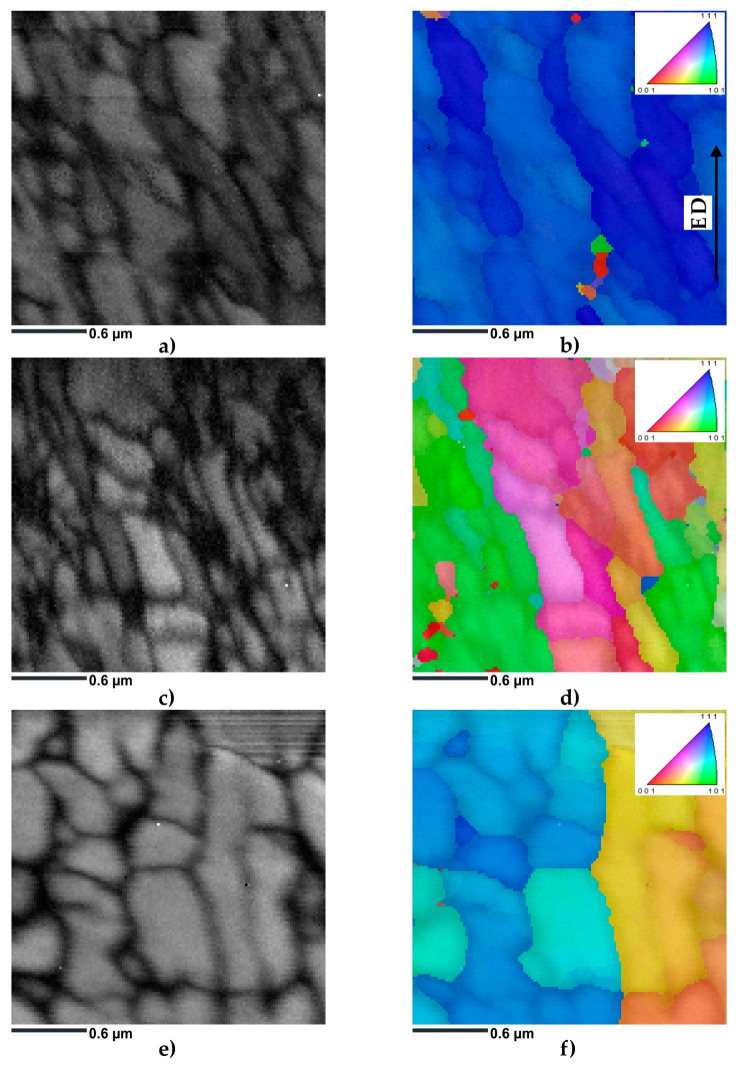
Result of the TKD EBSD investigation of ECAP-processed samples: (**a**) grayscale band contrast map of HT320E100 sample; (**b**) IPF-Y map of HT320E100 sample; (**c**) grayscale band contrast map of HT320E130 sample; (**d**) IPF-Y map of HT320E130 sample; (**e**) grayscale band contrast map of HT280E200 sample; (**f**) IPF-Y map of HT280E200 sample.

The microstructures of the ECAP-processed samples were first characterized using transmission Kikuchi diffraction mode in a scanning electron microscope. Figure 7 shows the EBSD TKD maps, which provide a comprehensive overview of the crystallographic orientations in a 2.5 × 2.5 µm region. The obtained images corresponded to three different samples subjected to ECAP processing at different temperatures: 100 °C, 130 °C, and 200 °C.

Figure 7a,c,e show that pattern quality was lower in certain areas and higher in others. This deterioration in BC quality was particularly evident in sample HT320E100. It was deduced that the areas with the lowest BC corresponded to the positions of the subgrain boundaries due to the interaction volume extending across the boundary, resulting in mixed diffraction patterns. In addition, a significant accumulation of dislocations near the Al/Si interfaces further contributed to the deterioration of the quality of the BC patterns. The SEM analysis of the HT320 sample showed that only a subtle change occurred in cell structure after heat treatment. Therefore, it was expected that these cell boundaries could hinder movement during the ECAP process, leading to their accumulation in these areas [44].

The quality of the BC also improved with increasing deformation temperature. As shown in Figure 7c, the interior of the subgrains exhibited a lighter color, suggesting a lower accumulation of dislocations. Furthermore, the BC quality at the subgrain boundaries was notably lower. Our overall observation indicated that the ECAP treatment at 130 °C resulted in reduced dislocation density.

Since it is easier to accumulate dislocations at lower deformation temperatures, the HT280E200 sample exhibited the highest BC quality (Figure 7e). This was due to higher ECAP temperatures that offered a more significant driving force for dislocation rearrangement, thus enhancing processes such as dynamic recovery [45].

Figure 7b,d,f show the IPF-Y TKD maps superimposed on the BC maps. The IPF-Y maps revealed parallel bands of elongated subgrains, which were aligned nearly parallel to the extrusion direction (ED). Remarkably, the sample deformed at 100 °C showed only minimal orientation differences, with most of the grains exhibiting the <111> crystallographic orientation (Figure 7b). The IPF-Y TKD map of the sample deformed at 130 °C showed a greater variety of crystallographic orientations in the studied region (Figure 7d). Predominantly blue and green colors were observed, which was consistent with the <101> and <001> orientations. Figure 7f shows the IPF-Y TKD map of the sample deformed at 200 °C, which revealed a shift towards a more equiaxed grain morphology. In addition, the predominant blue and green colors were consistent with the <101> and <001> orientations.

**Figure 8:**
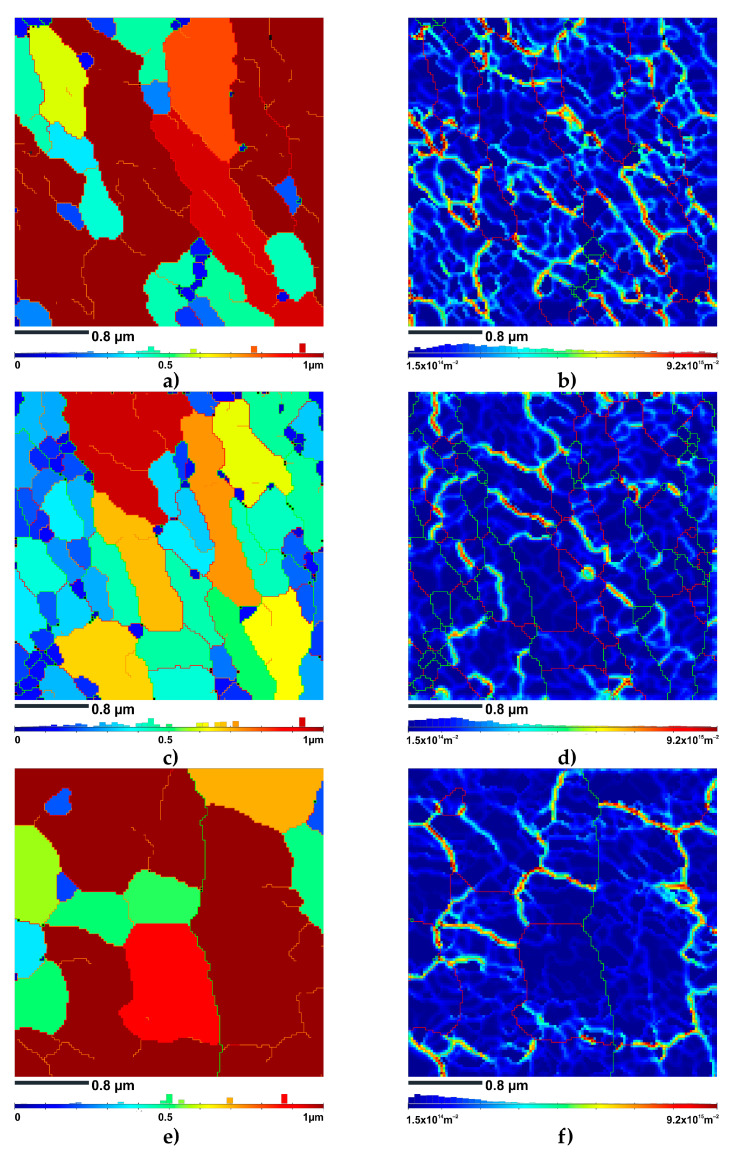
Result of the TKD EBSD investigation of ECAP-processed samples: (**a**) unique grain color map of HT320E100 sample; (**b**) GND map of HT320E100 sample; (**c**) unique grain color map of HT320E130 sample; (**d**) GND map of HT320E130 sample; (**e**) unique grain color map of HT280E200 sample; (**f**) GND map of HT280E200 sample.

The unique grain color maps obtained from the TKD experiments provided a comprehensive visualization of grain sizes within the ECAP-processed samples (Figure 8a,c,e). The maps were created based on the criterion that the minimum disorientation between adjacent points (grain tolerance angle (GTA)) was set at 2 degrees.

It should be noted that in the grain color maps, the grain boundaries characterized by a misorientation angle of 2 to 5 degrees are shown in orange, while the grain boundaries with a misorientation between 5 and 15 degrees are shown in red. Furthermore, the grain boundaries with a misorientation of more than 15 degrees are shown in green.

As shown in Figure 8a, the ECAP processing at 100 °C resulted in significant grain refinement. The unique grain color map revealed that the grain sizes varied from 0.4 to 0.8 µm, with an average of approx. 0.47 µm. According to the statistical data listed in Table 1, the LAGBs of the HT320E100 sample, characterized by misorientations below 15 degrees, accounted for approx. 88% of the total boundary fraction. In contrast, the HAGBs, characterized by misorientations greater than 15 degrees, accounted for approx. 12% of the total boundary fraction.

A remarkable fraction of grains ranging in size from 0.15 to 0.35 µm was observed in the HT320E100 sample. The grains possessed a distinct blue color on the unique grain color map (Figure 8c). Additionally, the average grain size was smaller than that of the HT320E100 sample, at approx. 0.27 µm. This could be attributed to two key factors: the influence of a heterogeneous microstructure and the relatively limited area subjected to TKD analysis. Hence, the LPBF microstructure was highly heterogeneous, resulting in grains with smaller sizes occurring in certain areas. The TKD analysis also provided limited information due to the small area studied.

Referring to the statistical data in Table 2, the LAGBs accounted for approx. 54% of the total grain boundary fraction. In contrast, the HAGBs accounted for approx. 46% of the boundary fraction. The observed increase in the fraction of HAGB may stem from the increased dislocation mobility at 130 °C, which allowed the dislocations to glide more easily, facilitating plastic deformation without the extensive formation of LAGBs.

Grains ranging in size from 0.7 to 0.9 µm were found in the HT280E200 sample. It had the largest average grain size of 0.7 µm among all the samples studied with TKD (Figure 8e). Additionally, the higher rate of dynamic recovery prevented the formation of dense LAGB arrays [46]. Therefore, the fraction of LAGBs accounted for only approx. 16% of the total boundary fraction, while the fraction of HAGBs accounted for approx. 84%.

Although the TKD analysis provided information regarding grain orientation, the grain boundary fraction and size of the area investigated were relatively small, at only 2.5 × 2.5 microns. While the small step size in EBSD TKD allowed for precise characterization, a quantitative analysis of the grain boundary characteristics would have required conventional EBSD techniques because of the limited area coverage in TKD. The results of the conventional EBSD studies were partially published [9,47] and contributed to a more comprehensive understanding of the microstructural evolution.

**Figure 9:**
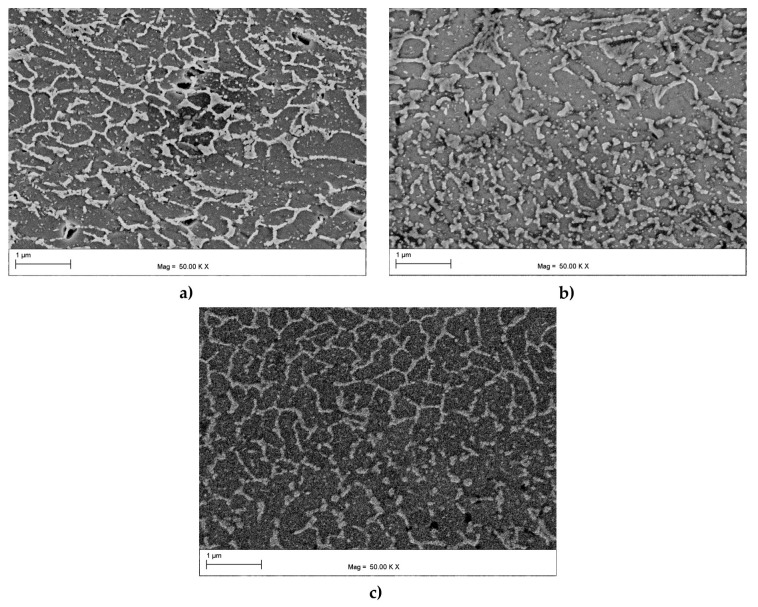
SEM microstructures of the AlSi10Mg alloy after ECAP processing: (**a**) HT320E100; (**b**) HT320E130; (**c**) HT280E200.

To analyze the dislocation distribution at the nanoscale, the GND maps were constructed based on the values of kernel average misorientation (KAM) (Figure 8b,d,f). The EBSD TKD map clearly showed significant strain partitioning during deformation, which occurred due to the high density of geometrically necessary dislocations (GNDs). Furthermore, the GNDs were heterogeneously distributed across the microstructures, with the majority located along the sub-GBs (LAGBs), especially those characterized by misorientation angles between 2 and 5 degrees. This indicated that GNDs were the source of LAGBs (GNDs rearrange into subgrain structures, reducing the strain distortion energy of the grain) [48]. Moreover, the GND map showed that the excessively small lattice curvature (~1°) within a subgrain was effectively compensated for by the nanoscale GND network. This nanoscale dislocation network was observed as light blue hues. Noteworthy, the overall dislocation density decreased significantly with increasing ECAP temperature (Table 1). This trend was consistent with the basic thermally activated mechanisms that govern the dislocation behavior [49]. At higher temperatures, the mobility of the dislocations increased due to the increased thermal energy. Consequently, the likelihood of dislocation interactions led to annihilation increasing, resulting in a reduction in the dislocation population. In addition, thermally assisted climb mechanisms became increasingly prominent, allowing dislocations to migrate out of their primary slip planes and contributing to the observed decrease in dislocation density. The decrease in GND density with increasing ECAP temperature can also be explained using the strain gradient theory. It is well established that the microstructure of an LPBF AlSi10Mg alloy is highly heterogeneous [50]. At the submicrometer level, it has a heterogeneous cell structure (Figure 5a). The cell boundaries are composed of the Si phase, which is much harder than the aluminum phase that fills the cell interiors. During plastic deformation, these domains contribute to the evolution of GND by generating the strain gradient [5]. According to mechanism-based strain gradient plasticity theory, the GND density is related to the strain gradient as follows:

(3) $$\rho_{GND}=(\frac{1}{\lambda^{G}})\frac{4\varepsilon }{b}$$

where $\rho_{GND}$ is the GNDs density, $\lambda^{G}$ is proportional to the domain separation (cell size), b is the length of Burgers vector, and ε is the strain level. Accordingly, one can expect the same GND densities for the same strain applied in an ECAP pass. However, an important factor affecting the storage of GNDs is the continuity of the eutectic Si network. It has been shown that in partially cellular microstructures, dislocations can penetrate the Si network, gradually decreasing the constraints they previously encountered [44]. As shown in Figure 5, the continuity of the Si network changed significantly during heat treatment. Subsequently, this network was further transformed during SPD processing. A comparison of microstructures presented in Figure 5c and Figure 9a revealed that the Si network was coarser and more disrupted after ECAP treatment at 100 °C than prior to deformation. Moreover, many point-like precipitates were formed within the α-Al cells, indicating that the ECAP treatment induced a high dislocation density, which enabled the nucleation of precipitates on dislocation cores [51]. Increasing the ECAP temperature to 130 °C further fragmented and coarsened the eutectic Si network (Figure 9b). Therefore, sample HT320E100, which had a more continuous Si network, exhibited a higher GND density. However, the HT320E130 sample, which exhibited a more interrupted and coarser cellular Si network, possessed a lower GND density. This was due to the partial discontinuity of the Si network, which allowed the dislocations to penetrate and traverse the cell interior more freely, resulting in less need to increase the GND density to compensate for the existing strain gradient. This effect was particularly visible in the HT280E200 sample, where the eutectic network underwent the greatest evolution (Figure 9c). Furthermore, the elevated ECAP temperature promoted recovery processes, leading to the rearrangement and annihilation of dislocations and effectively reducing GND density. Consequently, the grains contained fewer GNDs, as shown in Figure 8f.

#### 3.2.2. Transmission Electron Microscopy Analysis

**Figure 10:**
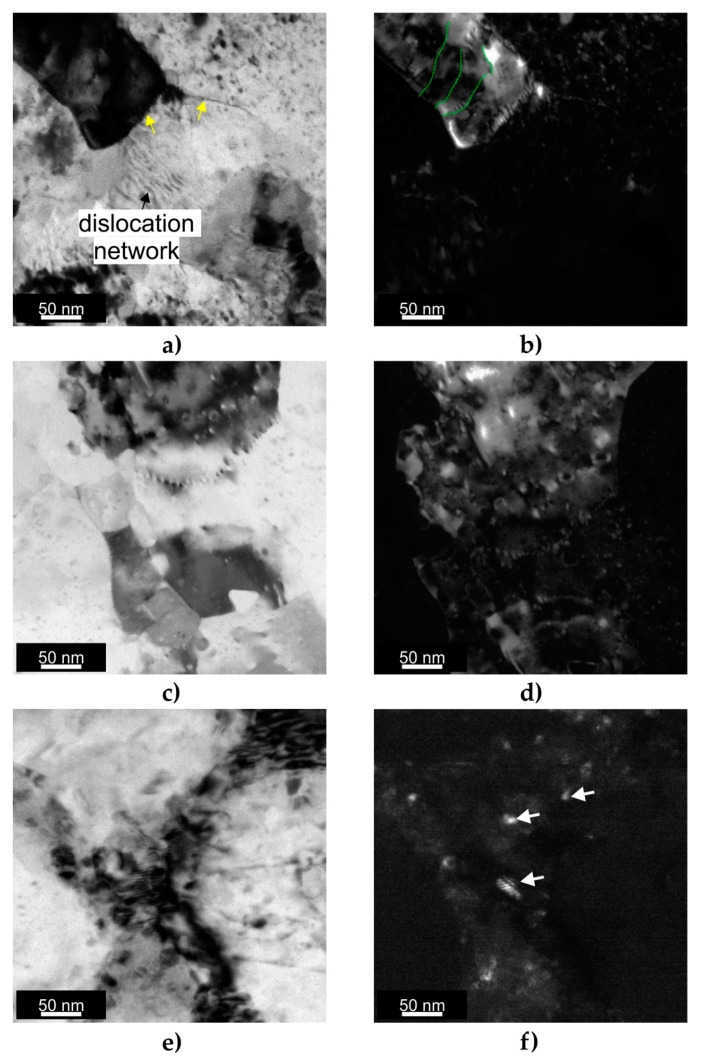
TEM characterization results: (**a**) BF TEM image of HT320E100 sample; (**b**) DF TEM image of HT320E100 sample; (**c**) BF TEM image of HT320E130 sample; (**d**) DF TEM image of HT320E130 sample; (**e**) BF TEM image of HT280E200 sample; (**f**) DF TEM image of HT280E200 sample.

TEM observations were performed to analyze the microstructures of the ECAP-processed samples in greater detail. Figure 10 shows the bright-field (BF) and the corresponding dark-field (DF) TEM images of three different ECAP-processed samples investigated. The bright-field TEM image shown in Figure 10a revealed that the minor crystallographic curvature was effectively compensated by dislocation structures (see yellow arrows). Moreover, the DF TEM image shown in Figure 10b displayed minor crystallographic orientation changes across individual subgrains (see green dotted lines), which may correspond to the nanoscale GND network revealed by the EBSD TKD analysis.

Figure 10c,d show the microstructure of the HT320E130 sample. As in the previous sample, the TEM image at high magnification revealed the dislocation of structures near the subgrain boundaries. Moreover, this image displays more subgrains (Figure 10d), suggesting that they were smaller than those in the HT320E100 sample. The DF images revealed subgrains with sizes ranging from 50 to 200 nm, which was consistent with the TKD analysis presented in the previous section.

The microstructure of the HT280E200 sample is shown in Figure 10e,f. The BF image (Figure 10e) displays the cell boundaries and the accumulation of dislocations near them, which appear as dark regions. The DF image confirmed that the cells consisted of small Si crystals, indicating a partial breakdown of the continuous Si network during deformation at 200 °C (see white arrows). The TEM observations were in agreement with the TKD EBSD results, which showed that the HT280E200 sample had the largest grain size among the ECAP-processed samples.

### 3.3. Sample Subjected to Multi-Axial Forging

#### Transmission Electron Microscopy Analysis

**Figure 11:**
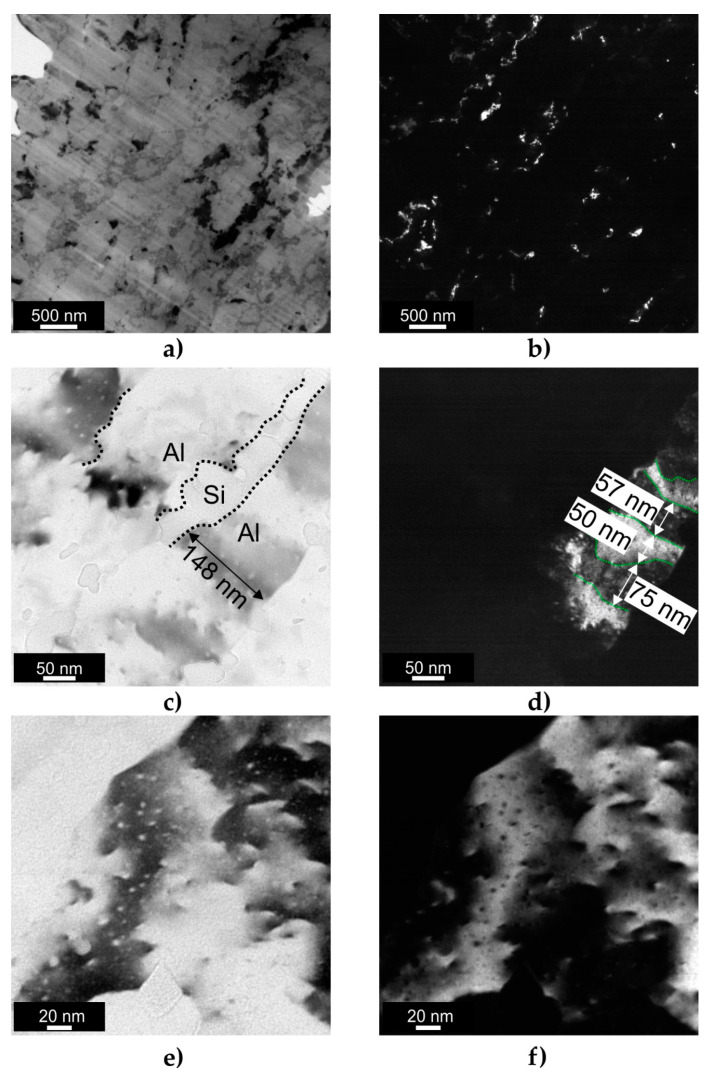
TEM images of the multi-axial forged AlSi10Mg sample: (**a**) BF TEM image showing the general microstructure; (**b**) DF TEM image showing the general microstructure; (**c**) BF TEM image taken at higher magnification revealing the subgrain size and Al/Si interface; (**d**) DF TEM image corresponding with that in Figure 11c; (**e**) BF TEM image revealing the dislocation accumulation inside subgrain; (**f**) DF TEM image corresponding to that in Figure 11e.

Figure 11 shows the TEM micrographs of the multi-axial forged AlSi10Mg sample. The DF TEM image shows a few subgrains with a width between 100 and 200 nm. Higher-magnification TEM images (Figure 11c,d) gave a more precise determination of the subgrain size. Figure 11c shows the microstructure of the MAF100 AlSi10Mg sample, which consisted of nearly parallel lamellar subgrains with a size of approx. 150 nm. Figure 11c shows the approx. 30 to 50 nm thick interconnected Si network that separated the subgrains. The DF TEM image shown in Figure 11d revealed the presence of relatively large differences within the lamellar subgrains (highlighted by a green dashed line), indicating that some regions had a slightly different crystallographic orientation than the surrounding area. This suggested that the lamellar subgrains were subdivided by a 50 nm to 80 nm thick nanoband structure.

According to the TEM results, the MAF100 sample exhibited the smallest subgrain size among the deformed samples. This was due to the MAF process creating highly heterogeneous microstructures in which the surface layer was deformed much more than the sample interior (Figure 4). This led to a more effective strain and statistically stored dislocation accumulation in the surface layer (Figure 11e,f), resulting in more pronounced grain refinement. In addition, this sample had a full-cellular microstructure before MAF deformation. Hence, during plastic deformation, the local strain gradient that exists between the cell interior and the Si cells promoted a much larger accumulation of GNDs, which were the source of LAGBs in the early stages of deformation. As the repetitive deformation was applied to the surface layer, these LAGBs were able to transform into HAGBs [52], which resulted in more pronounced grain refinement in the surface layer.

### 3.4. KoBo Sample

#### 3.4.1. Transmission Kikuchi Diffraction Analysis

**Figure 12:**
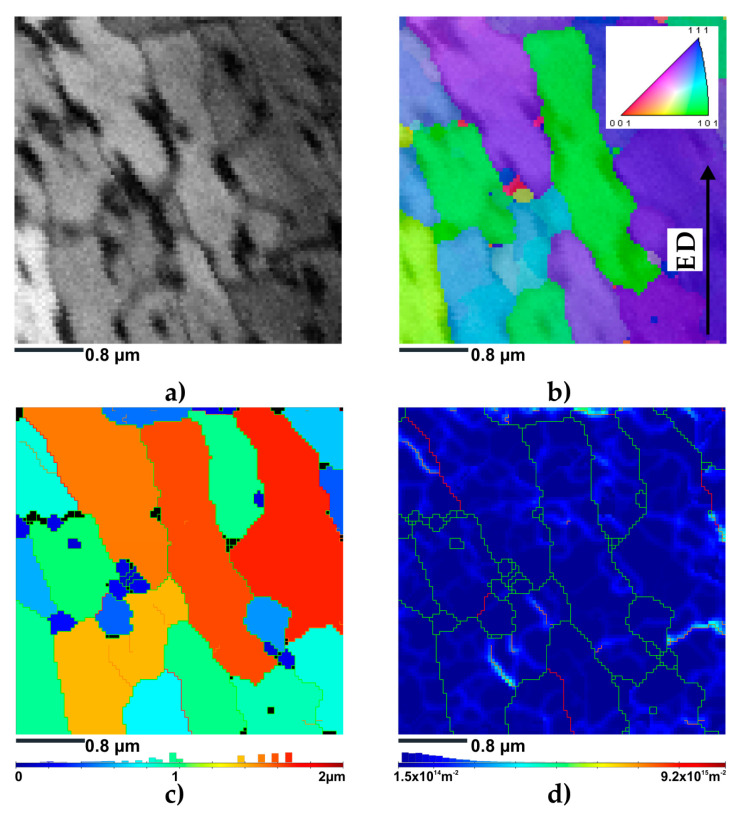
Result of the TKD EBSD investigation of KoBo sample: (**a**) BC map; (**b**) IPF-Y map; (**c**) unique grain color map; (**d**) GND distribution map.

Figure 12 shows the EBSD TKD map, which provides a comprehensive overview of the crystallographic orientations within an area of 3.6 × 3.6 µm. It should be noted that for the KoBo-processed sample, the step size for the TKD characterization was set at 40 nm, and a larger grain size was observed. As presented in Figure 12a, the band contrast map shows a relatively uniform contrast that only changes at the grain/phase boundaries, which implies a much lower dislocation accumulation in comparison with the ECAP-processed samples.

The IPF-Y map shows that the crystallographic orientation varied strongly in the investigated area (Figure 12b). This indicated the larger misorientation angles between the grains and subgrains. According to the unique TKD grain color map, LAGBs accounted for approx. 16% of the total grain boundary fraction. In contrast, HAGBs accounted for only approx. 84% of the total boundary fraction. The unique grain color map also confirmed that grains larger than 1 µm dominate in the investigated area (see grain size distribution in the bottom part of Figure 12c). The mean grain size was approx. 0.77 µm.

The GND map shown in Figure 12d displayed a much lower GND density of 4.35 × 10^14^ m^−2^. GNDs steadily accumulated near hot spots such as GBs, and especially near the boundaries having a misorientation angle between 2° and 5°. These GNDs accumulated at grain boundaries due to the slip discontinuities among grains, which created a strain gradient that required GNDs to maintain the geometric compatibility of the crystal [53].

#### 3.4.2. Transmission Electron Microscopy Analysis

**Figure 13:**
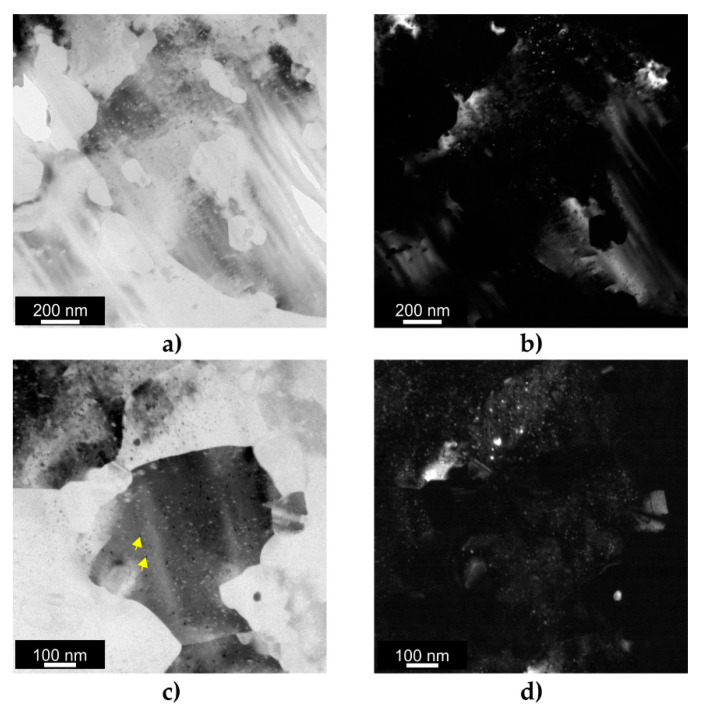
TEM images of the KoBo-processed AlSi10Mg sample: (**a**) BF TEM image; (**b**) DF TEM image; (**c**) BF TEM image taken at higher magnification; (**d**) DF TEM image taken at higher magnification.

To analyze the microstructure in greater detail, TEM analysis was performed. Figure 13 shows the BF along with the corresponding DF TEM images taken at two different magnifications. Since the contrast of dislocations depends on the diffraction condition and dislocations are invisible under certain orientations, the BF and DF TEM images revealed an almost dislocation-free structure of the KoBo-processed sample (Figure 13a,b). As shown in Figure 13c,d, the Kobo extrusion process resulted in the generation of point defects in the form of spherical clusters (see yellow arrows). Over the analyzed area, the grains exhibit relatively large contrast differences, indicating the presence of grain boundaries with high misorientation angles. Overall, the grains with sizes between 150 nm and 700 nm could be distinguished in the TEM images, which generally aligned with the TKD analysis.

## 4. Conclusions

In this article, an LPBF AlSi10Mg alloy was post-processed using different SPD methods. The microstructures were characterized via TKD and TEM. The main conclusions from this research are summarized as follows:

- The average grain size increased from ~0.3 to ~0.7 after ECAP processing in the temperature range of 100 °C–200 °C.
- The substantial enhancements in spatial resolution facilitated by TKD allowed for the revelation and characterization of a nanoscale GND network that compensated for the low lattice curvature in the ECAP-processed samples.
- The multi-axial forging resulted in the formation of the finest subgrain size. It was revealed that the lamellar subgrains were subdivided by a nanoband structure.
- The KoBo process resulted in the fabrication of an almost dislocation-free microstructure with an average grain size of approx. 0.77 µm.
- The KoBo-processed sample exhibited a lower geometrically necessary dislocation density than the ECAP-processed samples.
- The results of the microstructural investigations presented in this article indicated that the MAF was the most promising method for the preparation of UFG AlSi10Mg samples.

In summary, the comprehensive analysis presented in this article contributes to a greater understanding of the microstructural characteristics of the SPD-processed LPBF AlSi10Mg alloy, shedding light on the nature of grain boundaries and dislocation activities. These insights are crucial for tailoring material properties and optimizing processing techniques for various applications.

## Figures and Tables

**Table 1:** Summary of the microstructural characteristics of the AlSi10Mg samples.

Samples	As-Built	HT280	HT320
Average grain size, µm (GTA = 2°)	3.9	4.2	4.6
f_HAGBs_, %	79.8	86.4	86.3
f_LAGBs_, %	20.2	13.6	13.7

**Table 2:** Main microstructural parameters obtained from EBSD TKD investigation.

SAMPLE	HT320E100	HT320E130	HT280E200
ρ_GND_	1.35 × 10^15^ m^−2^	1.0 × 10^15^ m^−2^	9.67 × 10^14^ m^−2^
f_LAGBs_, %	88	54	16
f_HAGBs_, %	12	46	84

## Data Availability

The data presented in this study are available on request from the corresponding author.
